# Identifying older adults with frailty approaching end-of-life: A systematic review

**DOI:** 10.1177/02692163211045917

**Published:** 2021-09-14

**Authors:** Alex Hall, Elisabeth Boulton, Patience Kunonga, Gemma Spiers, Fiona Beyer, Peter Bower, Dawn Craig, Chris Todd, Barbara Hanratty

**Affiliations:** 1 National Institute for Health Research (NIHR) Older People and Frailty Policy Research Unit, School of Health Sciences, Faculty of Biology, Medicine and Health, The University of Manchester, Manchester, UK; 2 National Institute for Health Research (NIHR) Older People and Frailty Policy Research Unit, Population Health Sciences Institute, Newcastle University, Newcastle-upon-Tyne, UK

**Keywords:** Frailty, frail elderly, prognosis, palliative care, ROC curve

## Abstract

**Background:**

People with frailty may have specific needs for end-of-life care, but there is no consensus on how to identify these people in a timely way, or whether they will benefit from intervention.

**Aim:**

To synthesise evidence on identification of older people with frailty approaching end-of-life, and whether associated intervention improves outcomes.

**Design:**

Systematic review (PROSPERO: CRD42020462624).

**Data sources:**

Six databases were searched, with no date restrictions, for articles reporting prognostic or intervention studies. Key inclusion criteria were adults aged 65 and over, identified as frail via an established measure. End-of-life was defined as the final 12 months. Key exclusion criteria were proxy definitions of frailty, or studies involving people with cancer, even if also frail.

**Results:**

Three articles met the inclusion criteria. Strongest evidence came from one study in English primary care, which showed distinct trajectories in electronic Frailty Index scores in the last 12 months of life, associated with increased risk of death. We found no studies evaluating established clinical tools (e.g. Gold Standards Framework) with existing frail populations. We found no intervention studies; the literature on advance care planning with people with frailty has relied on proxy definitions of frailty.

**Conclusion:**

Clear implications for policy and practice are hindered by the lack of studies using an established approach to assessing frailty. Future end-of-life research needs to use explicit approaches to the measurement and reporting of frailty, and address the evidence gap on interventions. A focus on models of care that incorporate a palliative approach is essential.

**What is already known about this topic?**

- End-of-life services for people with cancer are well developed, but most older adults live with and die from non-malignant long-term conditions.
- Frailty is a health state characterised by a slow and gradual decline, which makes it difficult to identify when someone is entering the final 12 months of life.
- It is not clear how we can best identify older people with frailty approaching end-of-life, and whether/how identification and associated intervention improves their experiences and outcomes.

**What this paper adds?**

- This systematic review included three studies involving adults aged 65 and over explicitly identified as frail using established measures of frailty, and found that there are no widely accepted, evaluated ways of identifying when such people are moving into the final 12 months of life.
- The strongest evidence for identifying people with frailty who are approaching end-of-life came from a study of the electronic Frailty Index (eFI) in primary care in England, where a distinct trajectory in eFI scores in the last 12 months of life (low baseline, followed by a rapid rise, then plateau) was associated with increased risk of death.
- No evaluations (specific to people with frailty) were identified for the application of established clinical tools such as the Gold Standards Framework Prognostic Indicator (GSF), the Necesidades Palitivas (NECPAL CCOMS-ICO© Tool Version 1) or the Supportive and Palliative Care Indicators Tool 9 (SPICT)™.

**Implications for practice, theory or policy**

- Clear implications for policy and practice are hindered by the lack of evidence that relates to older adults explicitly identified as frail using established measures.
- Frailty is a well-established, distinct clinical entity with a series of measures developed in recent years, and future end-of-life research needs to use explicit approaches to the measurement and reporting of frailty.

## Background

Frailty, which has been described as ‘the most problematic expression of ageing’, is a health state in which people experience an accelerated decline in physiological reserve that leaves them less resilient to relatively minor stressor events.^1^ It is conceptualised via two principal models. The phenotype model posits that a person is frail if they present with three or more of five criteria (exhaustion, weight loss, weakness/loss of muscular strength, reduced gait speed and reduced energy/physical activity).^2^ The cumulative deficit model applies a Frailty Index (FI) containing 36 or more deficits; the number of deficits an individual has is divided by the total number in the index to give a score between 0 (no frailty) and 1 (extreme frailty).^3^ These two models are conceptually different and may be used complementarily. The phenotype model can facilitate immediate binary identification of the presence or absence of frailty without prior clinical evaluation, while the Frailty Index provides a summary of a comprehensive clinical assessment along a continuum.^4^

Two recent systematic reviews with meta-analyses have examined international prevalence and incidence of frailty respectively. Examining data from 1.75 million adults aged 50+ from 62 countries, 12% met the criteria for phenotypic frailty and 24% for the cumulative deficit model.^5^ Data from 120,000 adults aged 60+ from 28 countries show an incidence rate of 43.4 new cases per 1000 person-years.^6^ Frailty is drawing increasing attention in health policy and practice across the world.^7^ England is the first country to have introduced national policy on identification and stratification: since 2017, general practitioners are mandated to identify and support all patients aged 65 and over with moderate or severe frailty, and all those with severe frailty should receive annual medication and falls reviews.^8^ A widely used tool in English primary care is the electronic Frailty Index (eFI), which adopts the cumulative deficit model with 36 deficits and uses existing electronic health record information to quantify frailty.

Frailty is a strong predictor of mortality,^1^ but identification that someone with frailty is approaching end-of-life – commonly defined as the final 12 months^9,10^ – is difficult, because frailty is characterised by a slow and gradual decline.^11^ However, such identification is important, because people with frailty may have specific end-of-life care needs that should be carefully considered.^12,13^ Recent systematic reviews have focussed on the ability of frailty measures to predict mortality. An umbrella review of frailty screening tools (26 assessments and 8 indicators in total) found that the FI and gait speed were the most useful measures in routine care and community settings to predict adverse health outcomes including death.^14^ Another systematic review also found that the FI was a significant predictor of mortality over 2 to 19 years.^15^

Existing evidence from reviews confirms that the FI is a useful measure for predicting mortality over relatively long periods of time. However, it is unclear how to identify when patients already identified as frail are moving into the end-of-life phase. Use of the ‘surprise question’ (an approach in which clinicians reflect on whether they would be surprised if a particular patient were to die in the next 12 months) has been found to perform poorly for patients with frailty, and is not recommended for use in isolation.^16^ In the UK, the British Geriatrics Society offers guidance for situations of clinical uncertainty that highlights the need to identify and plan end-of-life care when recovery is not certain.^17^ Emphasis is placed on integrating geriatric medicine and palliative care, and planning for a number of possible outcomes, rather than a focus on identifying dying.^17^ The guidance recommends use of the Gold Standards Framework (GSF), the Necesidades Palitivas (NECPAL CCOMS-ICO© Tool Version 1) or the Supportive and Palliative Care Indicators Tool 9 (SPICT)™.^17^ The GSF and the NECPAL both start with the surprise question before considering indicators of frailty; the SPICT rejects the time frame used in the surprise question and offers an approach to holistic assessment and care planning in which prognostic uncertainty is accepted.

Methods for end-of-life identification and intervention need to be based on strong conceptual and empirical foundations, to ensure that they are a good use of health and care resources. Once identified, people should be offered appropriate care or effective interventions that will improve patient and carer experiences, quality of care and quality of life outcomes. It is therefore important to understand whether it is possible to identify people with frailty approaching end-of-life, and whether identification leads to appropriate and effective care. In this review, we aim to synthesise evidence on how to identify older people with frailty approaching end-of-life, and whether identification and associated intervention measurably improve their experiences and quality of life on standardised patient reported outcomes.

### Review questions

1. How can we identify older people with frailty approaching end-of-life?
2. Does identification and associated intervention with older people with frailty approaching end-of-life measurably improve their experiences and quality of life on standardised patient reported outcomes?

## Methods

We undertook a systematic review, which is reported according to the Preferred Reporting Items for Systematic Reviews and Meta-Analyses (PRISMA) guidelines.^18^ A protocol was registered on Prospero on 28 January 2020 (CRD42020462624).

### Searches

We developed and piloted a search strategy and carried out searches in the following bibliographic databases: MEDLINE (Ovid), Embase (Ovid), Healthcare Management Information Consortium (Ovid), Cochrane Library (Cochrane Database of Systematic Reviews and Cochrane Controlled Register of Trials), CINAHL (EBSCO) and Episkemonikos.

The strategy was designed by an experienced information specialist (FB) in collaboration with the two lead reviewers (AH and EB). It was based on combinations of terms relating to four concepts: (i) frail older adults; (ii) end-of-life; (iii) prognosis; and (iv) intervention. We combined concepts (i) AND (ii) AND (iii) for identification, and concepts (i) AND (ii) AND (iv) for intervention. These two combinations were merged and duplicates removed. We designed the search in MEDLINE, using MeSH headings and title and abstract key words (Supplemental File 1), and translated the strategy to other databases. The prognosis concept was informed by a tested filter published in the evidence synthesis methodology literature.^19^ Searches were conducted in December 2019, with no date restrictions.

To achieve more comprehensive results, we ran additional searches in MEDLINE, Embase and CINAHL using the MeSH subheading ‘mortality’ in place of the end-of-life string (ii). We initially did not include this term as the MeSH scope note states that it is used as a statistical concept referring to deaths in given populations. However, despite this we noted that some relevant records used this subheading, so we carried out supplementary searches that included it. The results of these additional searches were checked against the original searches and duplicates removed. The MEDLINE design is shown in Supplemental File 1. These additional searches were conducted in February 2020, again with no date restrictions.

### Review criteria

The review design drew upon PICOTS (Population, Intervention, Comparator, Outcomes, Timing, Setting) guidance for systematic reviews of prediction models.^20^ Review criteria are summarised in Table 1. Key criteria for inclusion were studies involving adults aged 65 and above, who were explicitly defined as frail (using the frailty phenotype model, the cumulative deficits model or any other established measure). The rationale for using explicit definitions of frailty was to acknowledge that although frailty overlaps with disability and comorbidity, it is a distinct clinical entity with a series of specific measures developed in recent years,^21^ and is under increasing focus within health systems worldwide. Studies with mixed populations were included if those meeting the age and frailty criteria were distinguishable.

**Table 1.** Inclusion and exclusion criteria.

	Inclusion criteria	Exclusion criteria
Population	Older adults (aged ⩾65) Sample explicitly defined as frail via one of the two major models of frailty: Fried’s frailty phenotype;^2^ Rockwood and Mitnitski’s cumulative deficits model.^3^ Studies where frailty is defined using other operational definitions	Older adults with cancer Studies using the term ‘frail’ as a generic adjective without justification or clear definition of frailty Older adults with major life-limiting diagnoses (e.g. dementia, heart disease, COPD, stroke, renal disease) who are not also explicitly identified as frail Studies with mixed populations where results from older adults who are identified as frail are indistinguishable from results from older adults who are not identified as frail
Interventions and Comparators	Prognostic prediction models for the identification of end of life in frailty. We defined a prognostic prediction model as one which estimates ‘the individualised probability or risk that a certain condition will occur in the future by combining information from multiple prognostic factors from an individual’;^22^ Prognostic prediction models linked to interventions (prediction model impact studies), through comparative studies where one group receives usual care provided without the model and the other has model predictions made available to guide decision-making; If there is a dearth of prognostic models, expand to include individual prognostic factors; Interventions where people with frailty identified as being at the end of life are treated with an intervention compared with usual care or another suitable comparator.	
Study design	Reviews or individual studies of prognostic prediction models Reviews or single comparative studies of prediction model impact studies Reviews or single comparative studies of interventions	Case studies, case series, non-controlled before and after studies, qualitative studies Abstracts and studies not in full form.
Outcomes	Prognostic models: model performance, including discrimination (the ability of the model to distinguish between patients needing end of life care and those who do not) and calibration (accuracy of predicted risk of end of life care, in terms of how the expected outcomes predicted from the model diverge from the observed outcomes). Interventions: patient outcomes (clinical outcomes, quality of life); health care utilisation and cost-effectiveness; patient experience.	
Time span of prediction	Individuals likely to die within 12 months^9^	Models that predict survival beyond 12 months Interventions for people expected to live beyond 12 months
Setting	All health care settings	

Key criteria for exclusion were studies where frailty was inferred by a proxy (e.g. long-term care residence), and studies that focussed on people who had a diagnosis of cancer, even if they were also identified as frail, because end-of-life care identification and subsequent care pathways for cancer are well developed.^22^ We also excluded case studies, case series and non-controlled before and after studies, to focus on the most robust types of evidence, and qualitative studies, as we were interested in performance of prediction models and effects of interventions.

**Figure 1.**
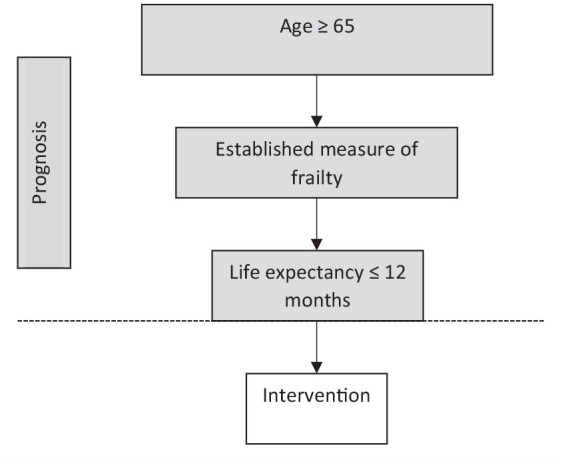
Application of key inclusion criteria.

We defined a prognostic model as being created from a combination of prognostic factors.^23^ If the prognostic model evidence base was very limited, we planned to expand the scope to include studies testing individual prognostic factors. We adopted the widely used time frame of the final 12 months of life for the end-of-life phase, which has been proposed in the palliative care literature^10^ and in England is reflected in National Institute for Health and Care Excellence (NICE) Quality Standards for end-of-life care.^9^ The application of the key inclusion criteria is summarised in Figure 1.

### Article selection

Records were screened in two stages: (i) titles and abstracts were screened for relevance by two researchers independently (AH and EB), with decisions compared and discussed to clarify and resolve inconsistencies; (ii) full texts of all records selected at stage one were retrieved and assessed against the review criteria by two researchers independently (AH and EB), with a third researcher resolving inconsistencies (PK or PB). During retrieval of full texts, other relevant texts not identified during database searches were also reviewed if potentially eligible.

We used EndNote X9 for reference management, the Rayyan web application^24^ for title and abstract screening and Microsoft Excel to record decisions on full-text screening.

### Data extraction, quality appraisal and synthesis

A data extraction form, using Microsoft Excel, was developed and piloted. Data were extracted as follows: authors and date; country; aim; design; setting; definition of frailty; population characteristics (age; other diagnoses); description of prognostic model or intervention; main findings; reported strengths and limitations; authors’ conclusions. Data were extracted by one researcher (AH) and checked by a second (EB).

To assess the strengths of conclusions that could be drawn from the evidence, we identified the following quality appraisal tools to assess risk of bias, for use as applicable:

- Systematic reviews: Risk of Bias in Systematic Reviews (ROBIS)^25^
- Prognostic studies: Prediction Model Risk of Bias Assessment Tool (PROBAST) ^26^; Quality in Prognostic Studies (QUIPS)^27^
- Randomised controlled studies: Cochrane Risk of Bias (RoB) 2.0^28^
- Non-randomised comparative studies: Cochrane Risk of Bias in Non-Randomised Studies-of Intervention (ROBINS-I).^29^

Quality appraisal was conducted by one researcher (AH) and checked by a second (EB or PK). We produced a narrative summary of findings.

We intended to synthesise findings using a narrative approach structured around our two research questions (performance of prediction models and effects of interventions). We planned to stratify results where relevant as follows:

- Populations distinguished between those with frailty who also have a major life-limiting diagnosis and those with frailty who do not have another diagnosis;
- Prognostication models and interventions grouped according to time span of end-of-life phase (e.g. 12 months, 6–11 months, 1–5 months, under 1 month).

## Results

### Selected articles, characteristics and quality appraisal

**Figure 2.**
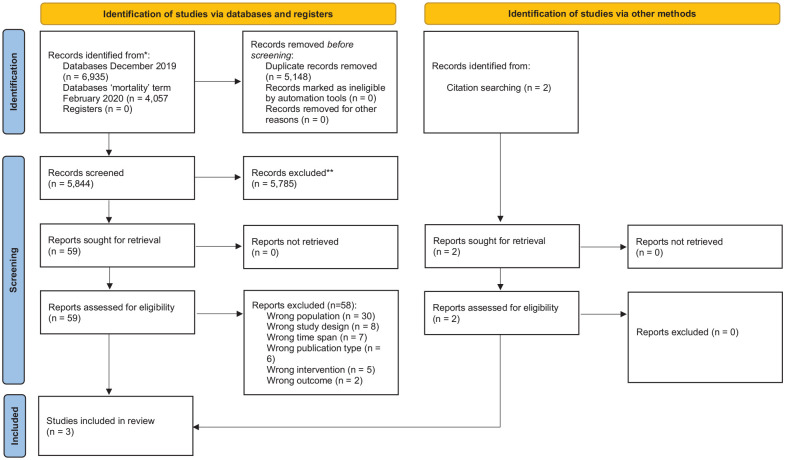
PRISMA flow diagram.

The PRISMA flowchart (Figure 2) shows that database searches yielded 5844 unique articles but that ultimately only three articles met the inclusion criteria.

Table 2 summarises the characteristics of the three included articles.^30–32^ These articles focussed on the identification of the end-of-life phase in people with frailty. One was a prognostic model study, from England. As the prognostic model evidence was so limited, we expanded our scope to include studies testing individual factors. The other two were prognostic factor studies, from Sweden and Japan respectively.

**Table 2.** Characteristics of included studies.

Study; country	Study type, design and setting	Aim	Measure of frailty	Population characteristics; *other diagnoses*	Prognostic tool/intervention description
Åhlund et al.^30^; Sweden	Prognostic (factor) Sub-study from a prospective controlled clinical trial Hospital (inpatient emergency medical care)	To analyse (1) the association between physical fitness measurements and 1-year mortality, and (2) the association between a preserved physical fitness during the first 3 months after discharge from emergency hospital care and 1-year prognosis	FRail Elderly Support researcH group (FRESH) screening instrument	*n* = 408 (56.7% female, mean age 85.7, range 75–99); 18 died during hospital stay therefore final total *n* = 390 in analysis. *Renal failure (87%), hypertension (71%), heart failure (40%), ischaemic heart disease (30%) and cerebrovascular disease (26%)*	Six-minute walk test (6MWT); handgrip strength test (HS)
Kamo et al.^31^; Japan	Prognostic (factor) 1-year prospective study Long term care (nursing homes)	To explore the relationship of coexisting severe frailty and malnutrition with all-cause mortality among the oldest old in nursing homes	Canadian Study of Health and Aging Clinical Frailty Scale (CSHA-CFS)	**Total:** *n* = 160, 88.75% female, mean 90.9 (SD 3.8). **Mild or moderate frailty** (CFS score 5–6): *n* = 37, 100% female, mean age 91.0 (SD 4.4). *Dementia (n* = *5), cerebrovascular disease (n* = *5), cancer (n* = *3), osteoarthritis (n* = *3), hypertension (n* = *10), heart failure (n* = *2), hip fracture (n* = *5), diabetes (n* = *1).* **Severe frailty** (CFS score 7+): *n* = 123, 85.4% female, mean age 90.9 (SD 3.7). *Dementia (n* = *51), cerebrovascular disease (n* = *33), cancer (n* = *6), osteoarthritis (n* = *9), hypertension (n* = *48), heart failure (n* = *12), hip fracture (n* = *17), diabetes (n* = *14).* **Co-existing severe frailty and malnutrition**: *n* = 75, 86.6% female, mean age 92.1 (SD 3.9). *Dementia (n* = *40), cerebrovascular disease (n* = *24), cancer (n* = *6), osteoarthritis (n* = *7), hypertension (n* = *39), heart failure (n* = *11), hip fracture (n* = *14), diabetes (n* = *6)*	Nutritional status assessed using Mini Nutritional Assessment – Short Form (MNA-SF); health status assessed through medical reports; overall mortality measured over 12-month follow up period via telephone/medical records
Stow et al.^32^; England	Prognostic (model) Longitudinal Primary care	To determine if changes in frailty measured by the eFI could be useful in primary care to indicate increased risk of dying and the need to consider palliative care	eFI	*n* = 26,928 (13,149 cases, 13,149 controls), 55.6% female, mean age at death 85.14 (SD 5.98) (cases)	eFI was calculated automatically by ResearchOne (extracts data from SystmOne clinical information system which hold records on half the UK population) at monthly intervals for 1 year, based on the information contained in each participant’s clinical record

A list of the 58 articles excluded at full-text review, with reasons, is provided in Supplemental File 2.^33–90^

Table 3 shows the risk of bias assessments of the three studies. All were all judged to be of low risk of bias.

**Table 3.** Risk of bias assessments of included studies.

Study	Stow et al.^32^		Åhlund et al.^30^		Kamo et al.^31^	
Overall RoB judgement	Low		Low		Low	
Appraisal tool	PROBAST		QUIPS		QUIPS	
RoB judgements by domain of tool	**Domain**	**Judgement** (RoB/Applicability concern)	**Domain**	**Judgement**	**Domain**	**Judgement**
	Participants	Low/Low	Study participation	Low	Study participation	Low
	Predictors	Low/Low	Study attrition	Moderate	Study attrition	Low
	Outcome	Low/Low	Prognostic factor assessment	Low	Prognostic factor assessment	Low
	Analysis	Low/Low	Outcome measurement	Low	Outcome measurement	Low
			Adjustment for other prognostic factors	Low	Adjustment for other prognostic factors	Low
			Analysis	Low	Analysis	Low

Given the paucity of studies, no formal synthesis or stratification was possible and we present each of the three studies in turn.

### Primary care: Clear trajectories in electronic Frailty Index scores

The prognostic model study by Stow et al.^32^ was a longitudinal case-control study of 26,928 primary care patients to determine if changes in eFI scores over a 12-month period could identify those at increased risk of dying. The authors found that repeat measurement of frailty using the eFI can support the identification of people with frailty who are approaching end-of-life. The study identified three distinct trajectories of eFI score. A small but clear proportion of the sample (2.2%) had a frailty trajectory characterised by a rapid initial rise from a low baseline, followed by a plateau. People in this group showed an initial increase of 0.022 eFI score per month, slowing from a baseline eFI of 0.21. This was associated with a mortality increase of 180% (odds ratio (OR) 2.84, 95% CI 2.34–3.45). This trajectory had 99.1% specificity and 3.2% sensitivity (positive predictive value 19.8%, negative predictive value 93.3%) for predicting individual mortality risk. Just under a quarter of the sample (21.2%) showed a pattern of moderately increasing frailty (eFI increase of 0.007 per month, baseline 0.26). This was associated with a mortality increase of 65% (OR 1.65, 95% CI 1.54–1.76). The largest group, around three-quarters of the sample (76.6%), showed a stable frailty profile, with an eFI increase of 0.001 from a baseline of 0.26.

### Emergency hospital care: Physical fitness

The prognostic factor study by Åhlund et al.^30^ investigated the predictive value of physical fitness among frail older patients who had received emergency hospital care. Frailty was assessed according to the FRail Elderly Support researcH group (FRESH) screening instrument, based on the phenotype model. Physical fitness measures used were to test aerobic capacity (using the six-minute walk test (6MWT)) and muscle strength (using handgrip strength test). Participants completed tests on three occasions: at baseline, at 3-month follow up, and at 12-month follow up.

Performance on both tests, at baseline and at 3-month follow up, was associated with 1-year mortality. On the 6MWT, people who walked less than 100 m in the index test were over three times more likely to die than those who walked over 200 m (hazard ratio (HR) 3.31, 95% CI 1.89–5.78, *p* = 0.001). People with low handgrip strength at index (<20 kg women; <30 kg men) had over twice the risk of dying than those with normal strength (HR 2.39, 95% CI 1.33–4.27, *p* = 0.003). Changes on both tests over the 0- to 3-month period were also associated with 1-year mortality, with those whose performance deteriorated having a poorer prognosis than those whose performance improved (6MWT: HR 3.80, 95% CI 1.44–10.06, *p* = 0.007; handgrip strength: HR 2.21, 95% CI 1.07–4.58, *p* = 0.032). A higher comorbidity burden (Charlson’s Comorbidity Index (CCI) score of ⩾8) or being male were associated with slightly higher mortality (6MWT: CCI ⩾8 HR 1.69, 95% CI 1.05–2.70, *p* = 0.03; male gender HR 1.69, 95% CI 1.19–2.38, *p* = 0.003; handgrip strength: CCI ⩾8 HR 1.70, 95% CI 1.07–2.68, *p* = 0.024; male gender HR 1.76, 95% CI 1.25–2.48, *p* = 0.001). However, severity of frailty and age were not associated with higher mortality.

### Nursing homes: Malnutrition or heart failure

The prognostic factor study by Kamo et al.^31^ investigated the predictive value of coexisting malnutrition and severe frailty among nursing home residents. Frailty was assessed by the Canadian Study of Health and Aging-Clinical Frailty Scale (CSHA-CFS), based on the cumulative deficit model, nutritional status was assessed using the Mini Nutritional Assessment – Short Form (MNA-SF), health status was assessed via medical reports, and mortality measured over 12-month follow up. Residents were stratified according to frailty level (mild/moderate or severe). The vast majority (*n* = 123/160) were severely frail, and nearly half (*n* = 75/160) had coexisting severe frailty and malnutrition. Cox regression analysis showed that after adjusting for age, gender and other diagnoses, coexisting severe frailty and malnutrition was significantly associated with mortality. Specifically, the risk was ten times greater, although there was a wide margin of uncertainty (adjusted HR 10.89, 95% CI 4.04–29.33, *p* < 0.0001). Across all levels of frailty, heart failure was also significantly associated with mortality; this risk was nearly eight times greater, but again, there was considerable uncertainty (adjusted HR 7.83, 95% CI 3.25–18.88, *p* < 0.0001).

## Discussion

### Main findings

Our systematic review looked for evidence on the identification of older people with frailty approaching end-of-life, and whether associated intervention improves quality of life outcomes. We found one prognostic model and two prognostic factor studies, but no intervention studies.

The first study provided evidence for use of the eFI in primary care to identify distinct frailty trajectories at end-of-life. This was a population-level study, not designed to produce practical tools for use with individual patients, and other research has shown that the eFI has low predictive value for mortality when used with individual patients 3 months prior to death.^80^ Two other studies identified potential prognostic factors that are common in later life and therefore unlikely to be of practical use in the specific context of frailty.

We found no studies evaluating the use of established clinical tools – for example Gold Standards Framework Prognostic Indicator (GSF); Necesidades Palitivas (NECPAL CCOMS-ICO© Tool) Version 1; Supportive and Palliative Care Indicators Tool 9 (SPICT)™ – with older adults who have been identified using an established measure of frailty. Similarly, we also found no studies evaluating any end-of-life care interventions for older people who had been formally identified as frail.

A key finding from our review is that established frailty measures have not been used in studies of end-of-life identification and intervention for frail older adults. Our inclusion criteria led us to exclude research in which the population had not been clearly identified as frail (*n* = 30/58 studies excluded at full-text review; see Figure 2 and Supplemental File 2). For example, Heppenstall et al.^56^ presented a study that aimed to develop methods for predicting 12-month mortality among long-term care residents, by testing the performance of geriatricians’ clinical judgement when provided with anonymous resident details (similar to the surprise question), and the performance of a logistic regression model applied to those details but without the geriatricians’ assessments. Both approaches performed only slightly better than chance (geriatricians: AUC = 0.64; regression model: AUC = 0.65). The authors asserted that their participants, as long-term care residents, constituted a frail population, though markers of frailty were not available. While it may seem intuitively plausible that such a population is frail, a recent systematic review and meta-analysis of the prevalence of frailty and prefrailty in nursing homes found that only around half of residents were frail according to validated criteria and definitions.^91^

In their cross-sectional study of prognostic indicators related to end-of-life trajectories, Amblàs-Novellas et al.^33^ reported that almost half of their cohort of 782 people with a positive NECPAL CCOMS-ICO test (indicative of likely need for palliative care) had advanced frailty. Palliative care needs were perceived to be low in this group; they did not share severity or progression criteria with people with diagnosed illness, and there were no distinct patterns in functional, nutritional and comorbidity indicators. The authors suggest a need for new conceptual models for end-of-life care in this population. Recent work with the NECPAL tool has attempted to identify prognostic indicators for 2-year mortality prediction, by review-of-reviews and expert consensus.^92^ However, only 3 of the 20 reviews included were concerned with older adults and frailty, and none of these reviews had clearly defined frailty. In the case of advanced cancer patients, a recent systematic review of 50 good-quality studies found that patient and informal carers have a wide range of context-bound unmet needs^93^; similar levels of pain and distress experienced by these patients are seen in people with frailty.^13^

Overall the lack of evidence for prognostic models and absence of prognostic markers in this population affirms the approach suggested by the British Geriatrics Society, prioritising formal assessment of needs over a search for prognostic indicators.^17^ Frailty research and clinical practice may have unintentionally assumed a very technical, biomedical interpretation of frailty at the expense of a more holistic approach to health and illness in later life that includes emphasis of positive attributes.^94^ The British Geriatrics Society resources direct clinicians towards advance care planning conversations, foregrounding that these conversations should begin as early as possible, be reviewed regularly and recognise parallel planning for scenarios of deterioration and recovery.

Two recent reviews have synthesised the literature on advance care planning for people who are described as frail,^95,96^ but the vast majority of this literature has not used established measures of frailty. Combes et al.^95^ conducted an integrative review of implementation of advance care planning with frail older people in the community; of the 11 intervention studies included, only one used an established measure of frailty, which was the study by Overbeek et al.^71^ However, this study did not use any prognostic indicator to identify participants at end-of-life phase; the assumption was based on the fact that participants had a mean age of 87, were in receipt of formal care and were frail.^72^ Overbeek et al.^72^ highlighted that they were surprised by a low mortality rate (10%) during their 12-month study period, and reflected that their participants were in better health than they had assumed. Hopkins et al.^96^ conducted a systematic review of advance care planning in acute inpatient settings, including 14 studies with adults aged 75 or over without a disease-specific focus. None of these studies used an established measure of frailty. Combes et al.^95^ reflected that use of long-term care residence as a proxy measure for frailty may have skewed findings away from people living in their own homes. Hopkins et al.^96^ called for better characterisation of study populations in frailty research as a priority. This call is not limited to frailty-specific research; routine measurement and reporting of frailty is largely missing in trials of novel pharmacological interventions for long-term conditions, but appears to be identifiable and prevalent in study populations from middle age and older.^97^

It is possible that intervention at end-of-life for people with frailty takes the form of lower-key interventions, such as small incremental enhancements to usual care, that are generally not captured in the published literature and are rarely evaluated in their own right, but nonetheless are likely to be of value. We did not find any studies that had evaluated such lower-key interventions for this specific population.

### Strengths and limitations

In this review, we adopted transparent, pre-specified criteria, including the need for studies to have applied an established measure of frailty. This is a strength for several reasons: first, it respects that frailty is a well-established, distinct clinical entity, with a series of assessment measures developed over recent years, second, it is aligned with the national health policy position in England that advocates routine measurement and monitoring of frailty,^8^ and third, it is commensurate with the recognition that people with frailty may have specific end-of-life care needs.^13^ However, these criteria necessarily excluded research that was relevant to but did not directly address the specific focus of the review (such as the advance care planning literature in which explicit assessment of frailty is lacking). We found a very limited literature meeting our specific criteria, and it should be noted that two out of the three studies meeting the criteria were studies of single prognostic factors, rather than multivariable models. We did not use a specific search filter for end-of-life search terms (e.g. Rietjens et al.^98^; this filter contains terms relating to bereavement which was not a focus of our review and we also wrote our search to exclude studies involving populations with cancer). However, as we screened around 6000 records retrieved from a robustly designed search we are confident that we have not missed any important literature.

## Conclusions

Clear implications for end-of-life policy and practice are hindered by the lack of evidence that relates to older adults explicitly identified as frail. Frailty trajectories, measures of physical fitness and assessment of malnutrition may all be helpful to indicate entry into the end-of-life phase, but the evidence is limited. There is also a paucity of evidence for appropriate interventions. Future research could helpfully adopt explicit measurement and reporting of frailty among study populations. In view of the challenges to identification of frailty and end-of-life, a focus on models of care that incorporate a palliative care approach within frailty is critical.

## Supplemental Material

sj-pdf-1-pmj-10.1177_02692163211045917 – Supplemental material for Identifying older adults with frailty approaching end-of-life: A systematic review Click here for additional data file. Supplemental material, sj-pdf-1-pmj-10.1177_02692163211045917 for Identifying older adults with frailty approaching end-of-life: A systematic review by Alex Hall, Elisabeth Boulton, Patience Kunonga, Gemma Spiers, Fiona Beyer, Peter Bower, Dawn Craig, Chris Todd and Barbara Hanratty in Palliative Medicine
