# Supplementary material for: Identifying older adults with frailty approaching end-of-life: A systematic review
Source: Palliat Med. 2021 Sep 14;35(10):1832–43. doi: 10.1177/02692163211045917 (PMC8637378; doi:10.1177/02692163211045917)
Supplement: sj-pdf-1-pmj-10.1177_02692163211045917 – Supplemental material for Identifying older adults with frailty approaching end-of-life: A systematic review [file sj-pdf-1-pmj-10.1177_02692163211045917.pdf]

Supplemental file 1: MEDLINE search strategies

The table below shows the full search strategy including the additional manipulation of the term 'mortality' conducted in February 2020. The original search strategy is in rows 1-26; the additional strategy is from row 27 onwards.

#	Searches	Results
1	Frail elderly/	10985
2	Frailty/	2077
3	frail*.ti,ab,kw.	20344
4	1 or 2 or 3	24440
5	Terminal Care/	27514
6	Hospice Care/	6332
7	Palliative Care/	52750
8	Terminally Ill/	6419
9	("end of life" or end stage or late stage or deterioriat* or declin* or dying or palliat* or life limiting or life-limiting or terminal*).ti,ab,kw.	966012
10	or/5-9	994189
11	(Stratification or Discrimination or Discriminate or c-statistic or "c statistic" or "Area under the curve" or AUC or Calibration or Indices or Algorithm or Multivariable).ti,ab,kw.	747655
12	ROC Curve/	55872
13	"Predictive Value of Tests"/	198365
14	observer variation/	41825
15	(predict* or scor* or observ*).ti,ab,kw.	5212233
16	or/11-15	5755012
17	(adaptive clinical trial or clinical trial or clinical trial phase i or clinical trial phase ii or clinical trial phase iii or clinical trial phase iv or comparative study or controlled clinical trial or evaluation studies or meta analysis or multicenter study or observational study or pragmatic clinical trial).pt.	2603782

18	exp epidemiologic studies/ or feasibility studies/ or pilot studies/	2565475
19	((intervention* or program* or strateg* or initiative* or project?) and (evaluat* or effect* or measur* or assess*)).ti,ab,kw.	1595158
20	or/17-19	5642691
21	4 and 10 and 16	1429
22	4 and 10 and 20	1842
23	21 or 22	2352
24	exp neoplasms/	3283100
25	23 not 24	2158
26	limit 25 to (english language and humans)	1674
27	mortality/ or hospital mortality/	80862
28	mo.fs.	561375
29	27 or 28	605629
30	4 and 29 and (16 or 20)	2103
31	30 not 24	1736
32	limit 31 to (english language and humans)	1659
33	32 not 26	1382
34	4 and 27 and (16 or 20)	702
35	34 not 24	677
36	limit 35 to (english language and humans)	646
37	36 not 26	545
38	4 and 28 and (16 or 20)	1671
39	38 not 24	1312
40	limit 39 to (english language and humans)	1260
41	40 not 26	1053
42	41 not 37	837

43	from 42 keep 1-837	837
44	Frailty/mo [Mortality]	191
45	44 and (16 or 20)	180
46	limit 45 to (english language and humans)	178
47	46 not 24	164
48	47 not 26	142
49	37 not 48	518
50	41 not (48 or 37)	722
51	33 or 48	1382

Supplemental file 2: studies excluded at full-text review

Study	Reason	Notes
Amblas-Novellas et al. 2016	Wrong population	Not already identified as frail
Amella 2003	Wrong study design	Not prognostic or intervention
Armstrong et al. 2010	Wrong publication type	Research letter
Arora et al. 2010	Wrong population	Not already identified as frail - 'vulnerable hospitalised elders'
Barclay et al. 2014	Wrong population	Not defined as frail
Brown et al. 2016	Wrong time span	> one year
Buchman et al. 2009	Wrong time span	> one year
Butcher et al. 2019	Wrong time span	> one year
Cardona-Morrell et al. 2015	Wrong study design	Review paper
Cardona-Morrell et al. 2017	Wrong study design	Narrative 'state of play'
Cardona et al. 2018	Wrong population	Not already identified as frail
Carey et al. 2008	Wrong population	Not clear that the population are all frail; not clear that people with cancer are disaggregated
Cole et al. 2019	Wrong population	Not defined as frail
Conroy et al. 2011	Wrong intervention	Not prognostic; interventions not specifically for end of life
Covinsky et al. 2003	Wrong population	Not clear that the population are all frail; not clear that people with cancer are disaggregated
Curtin et al. 2019	Wrong publication type	Conference abstract
Deng 2017	Wrong publication type	Conference abstract
Dent et al. 2016	Wrong study design	Review of frailty scales, not prognostic models
Ekerstad et al. 2017	Wrong population	Identified as frail but not as being in end of life phase
Espinoza et al. 2012	Wrong time span	> one year
Evans et al. 2016	Wrong publication type	Conference abstract
Giannini et al. 2007	Wrong intervention	Not prognostic; interventions not specifically for end of life
Glajchen et al. 2011	Wrong population	Cannot disaggregate cancer patients
Heppenstall et al. 2015	Wrong population	Long term care residence as proxy for frailty
Huang et al. 2007	Wrong population	Not defined as frail
Huijberts et al. 2016	Wrong population	Long term care residence as proxy for frailty
Iwata et al. 2006	Wrong population	Not defined as frail
Jakobsson et al. 2011	Wrong population	Long term care residence as proxy for frailty

Kaehr et al. 2016	Wrong population	Not already identified as frail
Kagansky et al. 2005	Wrong population	Frailty as synonym for old age
Kinley et al. 2014	Wrong study design	Implementation study
Landi et al. 2007	Wrong population	Not clear that the population are frail
Lunney et al. 2003	Wrong population	Long term care residence as proxy for frailty
Ma et al. 2013	Wrong population	Not already identified as frail
Mendonca et al. 2019	Wrong time span	> one year
Mukamel et al. 2004	Wrong population	Not clear that the population are all frail; not clear that people with cancer are disaggregated
Nouvenne et al. 2016	Wrong intervention	Biomarker study
O'Caoimh et al. 2014	Wrong population	Less than half the population clearly defined as frail
Overbeek et al. 2016	Wrong publication type	Conference abstract
Overbeek et al. 2018	Wrong intervention	Participants not clearly identified as being at end of life
Overbeek et al. 2019	Wrong intervention	Participants not clearly identified as being at end of life
Romoren et al. 2003	Wrong outcome	Not predicting entry into end of life phase
Salamanca-Balen et al. 2018	Wrong population	Frailty as synonym for old age
Sanchez-Rodriguez et al. 2019	Wrong population	Not clear that the population are frail
Stolz et al. 2019	Wrong population	Not identified as frail
Stow et al. 2018	Wrong population	Not already identified as frail
Sullivan et al. 1991	Wrong population	No access to article, but from 1991 which predates the conceptions of frailty in our criteria
Thomazeau et al. 2017	Wrong study design	Review; article in French
Todd et al. 2019	Wrong time span	> one year
van der Steen et al. 2005	Wrong population	Long term care residence as proxy for frailty
van Kempen et al. 2015	Wrong outcome	Not end of life
Vermeiren et al. 2016	Wrong population	Review; four papers of relevance but not identified as frail
Woods et al. 2019	Wrong publication type	Conference abstract
Wu et al. 2018	Wrong time span	> one year
Yash Pal et al. 2017	Wrong study design	Descriptive
Zasadzka et al. 2019	Wrong population	Not already identified as frail
